# Computer Security Incident Response Team Effectiveness: A Needs Assessment

**DOI:** 10.3389/fpsyg.2017.02179

**Published:** 2017-12-12

**Authors:** Rick Van der Kleij, Geert Kleinhuis, Heather Young

**Affiliations:** ^1^Behavioural and Societal Sciences, Department of Human Behavior and Organizational Innovations, Netherlands Organisation for Applied Scientific Research (TNO), Soesterberg, Netherlands; ^2^Centre of Expertise Cyber Security, The Hague University of Applied Sciences, The Hague, Netherlands; ^3^Technical Sciences, Department of Cyber Security and Robustness, Netherlands Organisation for Applied Scientific Research (TNO), Groningen, Netherlands

**Keywords:** incident handling, team performance, CSIRT, collaborative sensemaking, internal communication, CERT, team cognition

## Abstract

Computer security incident response teams (CSIRTs) respond to a computer security incident when the need arises. Failure of these teams can have far-reaching effects for the economy and national security. CSIRTs often have to work on an *ad hoc* basis, in close cooperation with other teams, and in time constrained environments. It could be argued that under these working conditions CSIRTs would be likely to encounter problems. A needs assessment was done to see to which extent this argument holds true. We constructed an incident response needs model to assist in identifying areas that require improvement. We envisioned a model consisting of four assessment categories: Organization, Team, Individual and Instrumental. Central to this is the idea that both problems and needs can have an organizational, team, individual, or technical origin or a combination of these levels. To gather data we conducted a literature review. This resulted in a comprehensive list of challenges and needs that could hinder or improve, respectively, the performance of CSIRTs. Then, semi-structured in depth interviews were held with team coordinators and team members of five public and private sector Dutch CSIRTs to ground these findings in practice and to identify gaps between current and desired incident handling practices. This paper presents the findings of our needs assessment and ends with a discussion of potential solutions to problems with performance in incident response.

## Introduction

Cyber threats pose major economic and national security challenges that need to be addressed (The White House, 2015). Over the past year, state actors and occupational criminals have caused many incidents or attempted to do so. In the Netherlands, the threat posed by these groups is big and has been growing over the past years (Dutch National Coordinator for Security and Counterterrorism, 2016). Given the increasing trends in cybercrime, it is necessary to protect the economy and nations’ critical infrastructure against these cyber threats. As it is simply impossible to completely prevent incidents, it is also critical to have the capacity to respond quickly and effectively when cyber security incidents occur (Cichonski et al., 2012).

The National Institute of Standards and Technology (NIST) defines an incident as “a violation or imminent threat of violation of computer security policies, acceptable use policies, or standard security practices” (Cichonski et al., 2012, p. 6). An example of an incident is an attacker who commands a botnet to send high volumes of connection requests to a web server, causing it to crash. In the Netherlands several organizations provide incident response services. Their services are performed for a parent entity, such as a corporate, governmental, or educational organization; a research network; or a paying client. Examples of incident response teams in the Netherlands are: FoxCERT, DefCERT, Northwave-CERT and SURFcert.

NIST identifies several benefits of having an incident response capability (Cichonski et al., 2012, p. 6). An important benefit is that this capacity helps to respond to incidents in a systematic manner (i.e., following a consistent incident handling methodology). This helps to maximize the chance of taking the appropriate actions to handle the incident. Moreover, incident response capability helps organizations minimize the consequences of incidents. For instance, by adequately handling the incident, loss or theft of information and disruptions of work processes caused by incidents could be minimized. Yet another benefit is the ability to learn from incidents. By using information gained during incident handling an organization or state actor is able to build stronger protection from future intrusions and, at the same time, is better prepared for handling future incidents.

Computer security incident response teams (CSIRTs) play an important role in the Netherlands in responding to incidents and achieving the aforementioned benefits. Incident response teams can be formalized, such that performing incident response is its major function. These teams can also be more *ad hoc* in nature, in that members are called together to respond to an incident when the need arises^1^. Usually, these members work in IT-departments within the organizations themselves. After an incident has been detected, one or more team members, depending on the familiarity and magnitude of the incident and availability of personnel, will initially handle the incident. Ideally, the team analyzes the incident data, determines the impact of the incident, and acts appropriately to limit the damage and restore normal services (Chen et al., 2014).

The CSIRT’s success depends on many factors, such as the technical resources at their disposal and team members’ level of knowledge and skills. In addition to these factors, a team’s success also depends strongly on the participation and cooperation of individual CSIRT members and other individuals, teams, and departments within and outside the organization (Cichonski et al., 2012). Hence, teamwork is of the utmost importance in incident handling. Teams have the potential to offer greater adaptability, productivity, information processing capacity, and creativity than any one individual can offer (Gladstein, 1984; Hackman, 1987; Salas et al., 2005). Moreover, teamwork is vital to transforming individual members’ disparate incident knowledge into a shared awareness of the evolving situation (Rajivan and Cooke, 2017).

Not only is teamwork within one’s own incident response team important. The nature of contemporary threats and attacks makes it more important than ever that incident response teams work together with other actors during incident response as well (Cichonski et al., 2012; Tetrick et al., 2016). It is necessary to build relationships and establish means of communication within the incident response team, with other groups within the organization (e.g., human resources, legal departments) and with external stakeholders (e.g., other incident response teams, law enforcement, IT-department of the customer organization, software vendors) (Cichonski et al., 2012; see also, Hámornik and Krasznay, 2018). In practice, many informal networks exist, in which, for instance, members responsible for the technical details of incident response share strategies and methods for mitigating attacks. During attacks, these networks enable team members to coordinate incident response with operational colleagues at partner organizations. Moreover, during the same incident, team managers may seek advice and additional resources for successfully responding to the incident at the government level. Ideally, CSIRTs share threat, attack, and vulnerability information with each other so that each organization’s knowledge benefits the other (Cichonski et al., 2012).

From the general teams literature it is known that teams are not easily implemented, that the creation of a team of skilled members does not ensure success, and that teamwork does not just happen. In fact, many teams never reach their full potential, and many fail altogether (Hackman, 1987; Salas et al., 2005). CSIRTs are not just ordinary teams. As we have argued, these teams often have to work on an *ad hoc* basis, in close cooperation with other parties, and in crisis situations. It could be argued that especially under these working conditions CSIRTs would be likely to encounter problems. Failure of incident response teams can have far-reaching effects on their respective organizations and the client organization (e.g., low speed to solution, low time to identification, high number of errors, high costs, and low ability to remove threat). This raises the question: what ensures success in computer security incident response teams? Surprisingly, efforts to improve teamwork and collaboration within cybersecurity organizations have been minimal (Rajivan and Cooke, 2017). This paper focuses on identifying areas for improvements and on potential solutions to problems with team performance in incident response.

## Materials and Methods

A needs assessment was conducted to identify gaps between current and desired incident handling practices. A needs assessment is a systematic process for determining and addressing ‘gaps’ between current results and desired result; or ‘wants’ (Kaufman et al., 1993; Watkins et al., 1998). An additional goal of the needs assessment described in this paper was to provide directions for future research and development for improving the effectiveness of team performance in incident response.

To eventually provide for an effective solution strategy we constructed a needs assessment model consisting of four assessment categories: Organization, Team, Individual, and Instrumental. Organization needs pertain to incident handling behavior or tangible outcomes, such as time to identification, or ability to remove threat. Team performance needs pertain to the state of the team or level of team performance required for satisfactory functioning, such as team structure. Individual needs pertain to individuals’ attitudes about the organization or themselves, such as job satisfaction or competences. Instrumental (or technical) needs are interventions or products that are required to obtain a satisfactory level of functioning.

An important step in needs assessment is gathering appropriate and sufficient data. There are many approaches identified in the literature for completing an assessment. We chose a multi-method data-triangulation technique relying on literature reviews and survey data (see also, Watkins et al., 1998). For the literature review a three step structured approach was used to determine the source material for the review as suggested by Webster and Watson (2002). The first step was to search relevant journal databases and the web for identification of relevant articles. The Scopus library database and Google Scholar search engine were used to get published content, but also to find content not yet indexed by library databases. The search terms *challenges*, *needs*, *issues*, *CERT*, and *CSIRT*, were used on Scopus database and in our web search. The search terms were used singular and in combination for all fields (including title, abstract, and full text). As a second step, backward reference searching was performed on the citations for the articles that were identified in step one to determine prior articles. Forward reference searching was used as a third step to identify articles citing the key articles identified in the previous steps. The library database search, the web search and reference searching methods resulted in 31 relevant contributions, which are cited throughout this paper. Hence, in these contributions challenges or needs are discussed that could hinder or improve the performance of incident response teams.

Furthermore, a selection of Dutch public and private cyber security organizations were contacted to participate in an interview. This resulted in five semi-structured interviews with senior management of public and private sector CSIRTs. The interviews were used to validate our findings from the literature search. The protocol that we used was approved by our institutional ethics committee, as was our study design. These five CSIRTs included a governmental coordination center, internal and commercial CSIRTs, which were all licensed at the time to use the name CERT by Carnegie Mellon University. The interviews included questions about challenges in incident handling that were identified in content from library databases and other sources. An example question is: “We identified several challenges in incident handling in literature. Could you have a look at these issues and explain to us which of these issues apply to your practice and why”? Each interview took approximately 2 h to complete. All subjects gave written informed consent in accordance with the Declaration of Helsinki to participate in the interview and to publish the research in scientific outlets. Transcripts of these interviews were made afterward by the interviewers and were sent to all the interviewees who agreed to check them (cf. Rowley, 2012).

## Results

As mentioned, we constructed a needs assessment model for categorizing needs and wants that play a role in incident response consisting of the four assessment groups described earlier. This model is composed of organizational categories (organization, team, and individual) and one instrumental or technical category. Central to this is the idea that wants or challenges can have an organizational, team, individual, or technical origin or a combination thereof (cf. Security Incident Management Maturity Model [SIM3]) (Stikvort, 2015). In **Table 1**, for each of these four categories, we indicate which four needs and wants were most frequently indicated by the interviewees. Discussion takes place in subsequent paragraphs and in the “Discussion” section.

**Table 1:** Overview of needs and wants of Incident Response Teams.

Organization needs
• Coordination and sharing information with outside parties
• Organizational and incident learning
• Measuring the effectiveness of incident handling
• Collaborative problem-solving capacity and shared incident awareness
**Team performance needs**
• Information sharing and decision making across personnel shifts and handoffs
• Work within a larger (multiteam) system consisting of multiple interacting teams, including IT personnel from customer
• Keeping everybody informed and staying informed, especially when working distributed
• Shared team knowledge: Information about the roles and expertise of each team member, including members of outside parties involved in the incident handling process
**Individual needs**
• Getting and retaining good skilled personnel and acquiring relevant competences
• Deciding on when to escalate an incident
• Ethical and legal aspects of the work
• Dealing with work load variations: managing peaks and underload
**Instrumental needs**
• Estimating the initial impact and risk of cyber security incidents
• Need for better interpersonal communication tools, especially during larger incidents
• Providing good and structured reports of incidents
• Creating useful (visual) overviews at any particular point in time for a different audience (e.g., customer, management, and colleagues)

### Organization Needs

In today’s networked working environment resolving incidents typically requires social interactions, information sharing and collaboration between organizations. A CSIRT does not operate in a vacuum but within the context of a complex sociotechnical system (Rajivan and Cooke, 2017). The most important cooperation partners for CSIRTs are fellow teams (West-Brown et al., 2003). Other teams could provide information in support of handling the incident. The interviews made clear that sharing information is not as easy as one might think. Many web-based tools are available to support information sharing between organizations. However, the extent to which teams are able or willing to exchange information and to cooperate on confidential issues depends on any existing trusted relationship they may have with each other. Formal (written) agreements between teams or organizations to exchange information and (national) platforms for trusted CSIRT communication are often in place to manage trusted information exchange. Although CSIRTs may benefit from trusted communication, our interviews confirmed that there are strong inhibiting factors as well (see also, Silicki and Maj, 2008; Hellwig et al., 2016; Tetrick et al., 2016; Rajivan and Cooke, 2017). For example, commercial CSIRTs’ upper management generally do not like their team’s resources spent on ‘outside’ parties, such as competing CSIRTs (see also, West-Brown et al., 2003). Moreover, client companies are usually not interested in information sharing with the CSIRT community (Hellwig et al., 2016). Companies are motivated to protect their reputation as a cyber safe and secure organization. It is important that the threats they face are quickly and quietly solved. Sharing information about a compromised system may put them in a vulnerable position (Tetrick et al., 2016, p. 113).

Incident learning can be seen as the process of creating, retaining and transfer of knowledge regarding incident handling within the organization. CSIRTs seem to struggle with incident learning (cf. Tøndel et al., 2014). The practice of incident response frequently does not result in the improvement of strategic security processes such as policy development and risk assessment. Ahmad et al. (2012) add to these findings that when a post-incident review process does take place it usually focuses “on ‘high impact’ incidents rather than ‘high learning’ (i.e., potentially useful incidents from a learning perspective), incidents and ‘near misses”’ (p. 1). So called false positives and threat hunts with negative outcomes are often not documented in ticketing systems by analysts. Hence, there is the risk for analysts, in the already information overloaded cyber security environment, to respond to weak signals that have already been cleared as non-significant by fellow team workers.

Another important organizational need is the ability to measure the effectiveness of services and the effectiveness of the team itself. Relatively little is being done by the companies interviewed to measure how effective their incident response services are in handling incidents. This is further hindered by the fact that there are hardly any metrics available to objectively measure the effectiveness of incident handling (cf. Bada et al., 2014; see also Wiik et al., 2006). To quote one of the interviewees: “we do not know how effective our services are.” Granåsen and Andersson (2016) found that a combination of technical performance measurements and behavioral assessment techniques are needed to effectively assess team effectiveness. Many technical metrics are already regularly and successfully used to assess incident management, such as speed to solution, time to identification, number of errors, costs, and so forth. These are important for the lessons learnt phase of incident response (see also Tøndel et al., 2014). Other indicators, such as incident rates over time and mean time to repair, could also be beneficial to achieve a better view of the origin of incidents, which system domains and particular applications are involved, and so forth. What is sorely lacking, however, are behavioral metrics to assess processes such as team performance and cooperation.

Yet another organizational level need that we would like to mention is the ability to be better at the process of assessing the incident. It is often difficult to make a good assessment of the incident at the start of the incident handling process. CSIRTs often face unfamiliar problems and have to make sense out of a seemingly unstructured situation (see also, Wu et al., 2013). The interviewees often referred to the iceberg metaphor in describing their work, in which the greater part of the iceberg is hidden under water so the part that you see at the onset of the incident is much smaller than the part that is hidden. An important CSIRT task is to find out how big the ‘iceberg’ actually is, according to the interviewees. It would be interesting to find out whether some sort of procedural support could be envisioned in aid of this process to benefit CSIRT performance.

### Team Performance Needs

Cyber incident handling errors often occur during handoffs (Steinke et al., 2015). Handoffs are the moments during which work is passed from one person to another person, for instance between team personnel shifts. However, handoffs can also take place within shifts, between individuals, people and technology, an individual and a team, and one team and another team, creating multiple places for errors and mistakes in the process. When incidents arise, there is a need for team members to be able to effectively communicate all information associated with those incidents throughout the team. For example, when the incident falls outside a team member’s ability and it needs to be handed over to colleagues with greater ability or familiarity with the incident at hand (cf. Tetrick et al., 2016, p. 80). In the interviews the fact was acknowledged that handoffs can be especially challenging in large scale incidents, spanning several days and involving multiple parties.

Another important need is the ability for CSIRTs to work within a larger (multiteam) system, which consists of multiple interacting and closely connected component teams, from within the own organization, but sometimes including IT personnel from other organizations, such as client organizations (Chen et al., 2014; Tetrick et al., 2016, p. 10). To quote Chen et al. (2014, p. 62), “each team in the system has its own domain expertise, jargon, demographics, culture, structure, and temporal dynamics. In such contexts, team members must be comfortable working across team boundaries to collaborate and share information. Each team also brings its own expertise to the system, and all involved teams must work together effectively to accomplish a shared goal” (see also, Van der Kleij et al., 2011). A specific component team can excel in teamwork but still fail to resolve cybersecurity incidents due to mistrust or a lack of communication among individual component teams (Tetrick et al., 2016, p. 10).

Chen et al. (2014) illustrate the difficulties multiteam systems face with an example of a provincial reconstruction team (PRT). PRTs are teams staffed with smaller military and civilian units helping local communities in instable countries with reconstruction work. Chen et al. (2014) describe an example in which, due to communication breakdowns between teams in the PRT, time and resources were wasted, delaying the handing out of critical medical aid. This highlights the necessity of communication and coordination across different system units. As CSIRTs are also part of a multiteam system, often working on highly important tasks under time pressure, they are likely to face information-sharing challenges similar to those described in the PRT example. Effective between-team coordination and communication are needed for incident response teams to accomplish tasks efficiently and effectively.

Another need mentioned in the interviews is the ability to maintain a shared understanding of the incident and of the ongoing and planned tasks of fellow workers, especially in a geographically distributed setting. Maintaining an ongoing awareness of events and each other’s endeavors is essential to achieving the coordination required for collaborative action (Van der Kleij and Te Brake, 2010). Incident response team members need to keep up with information about how particular tasks are progressing, what fellow workers are doing, who is communicating with whom, and so forth. Team members need knowledge of what other team members are doing. Without this knowledge it becomes difficult, or even impossible, to engage in coordinated teamwork (Van der Kleij and Te Brake, 2010). A complicating factor is that incident response team members often work from different locations, that is, team members often work in geographically distributed teams. It is more difficult to monitor fellow team members’ activities and pick up relevant information or cues in the absence of face-to-face communication. Consequently, team members must often work in an environment without any signals to indicate that a team member is busy, experiencing technical difficulties, stressed, dealing with unusual or unexpected circumstances, and so forth (Van der Kleij et al., 2009).

Most interviewees acknowledge that incident response teams are often formed on an *ad hoc* basis. However, this does not mean that members do not know each other or are chosen randomly. Members are chosen based on expertise from a fixed pool of CSIRT employees. Notwithstanding the fact that members often have a shared understanding of how their expertise and roles fit together, the development of shared team knowledge is recognized as being beneficial to incident handling. Shared team knowledge includes information about the roles and expertise of each team member, including members of outside parties that are involved in the incident handling process (Steinke et al., 2015). With shared team knowledge, team members can more successfully coordinate their work.

### Individual Needs

Although the need of finding and retaining good, skilled personnel and training personnel is certainly not new (see West-Brown et al., 2003), it remains relevant according to the parties we have spoken to. The expectation is that shortages of highly skilled personnel on the market will only increase in the years to come (Dutch National Coordinator for Security and Counterterrorism, 2016). The staffing of CSIRTs requires a blend of technical and team skills (cf. Chen et al., 2014; Steinke et al., 2015). Individual members often have to work with other team members and people from outside the parent organization. This means that several collective-level competences are important, including information sharing skills, collaboration skills, and a preference for working with others (Chen et al., 2014). The companies we have spoken to all confirm that there are often gaps in employees’ social skill sets that require additional training, the hiring of additional personnel to interface between the more technical skilled personnel and customers, or that need to be taken into account when staffing smaller incident handling teams.

Computer security incident response team work is mostly individual until a non-routine or unfamiliar incident occurs (Chen et al., 2014). Tasks typically originate at the individual level, wherein one member identifies a potential incident and must decide whether to involve other team members to mitigate the incident, or to hand it over to a more experienced colleague. For instance, the interviews revealed that in incident response the intake of security incidents is usually performed by a low-level cybersecurity employee operating a helpdesk or call center (see also Tetrick et al., 2016, p. 80). This member then has to decide whether mitigating the incident requires a handover or assistance from other more experienced members. Herein lies an interesting difference with other types of crisis management teams, such as firefighting teams, in which tasks usually begin at the team level, and individual members are not burdened with making decisions about when to initiate collaboration (Chen et al., 2014). Moreover, under some circumstances, such as when time pressure is involved, individual members may erroneously conclude that the incident is familiar to them, and, consequently, fail to seek help from others (Tetrick et al., 2016, p. 83). A task analysis performed by Chen et al. (2014) suggests that when an event requires the other members’ assistance, and the individual members decide to involve other people, several collective-level competences become important, including collaboration skills and information-sharing skills. Chen et al. (2014) argue that the work is multilevel in nature, comprising an individual and a collaborative level. This suggests that team members should possess the skills to know when and how to escalate events. Moreover, a set of information-sharing and collaboration norms should be established according to Chen et al. (2014, p. 65) that “let team members accurately determine when an event requires other teams’ or multiteam members’ involvement.”

A concern that was voiced by the interviewees is about integrity and consequences of ethical considerations during work on mental wellbeing of employees. Team members sometimes encounter illegal material on client databases or are obligated to report findings to the government, for instance regarding security breaches resulting in, among others, theft, loss or misuse of personal data. This could potentially damage the reputation of the client organization, resulting in an ethical dilemma for the incident response team member: reporting the incident or findings to legal authorities or telling the client organization to do so and trusting them to take appropriate measures.

Data breach notification laws are sometimes also responsible for peaks in workload that need to be managed by the team members accordingly. Both private and public organizations processing personal data are obliged to report any security breaches resulting in theft, loss or misuse of personal data to the Dutch data protection authority^2^. The data breach has to be reported without undue delay and if possible not later than 72 h after the discovery of the data breach. This means that incident response teams, when theft, loss or misuse of personal data is involved or is suspected to be involved, have a limited time frame to discover what is lost and to prevent further loss of (personal) data. Hence, incident handling teams often have to work under time pressure. On top of that comes the fact that teams often have to work at night or during weekends. In an effort to safe money, customers choose to seek support only after they themselves have failed to handle the incident successfully. Often this means that reporting of the incident takes place at the end of the work day, or even at the end of the work week, leaving the incident response teams the night or the weekend to work on the incident at hand.

### Instrumental Needs

From the interviews it became clear that CSIRTs have various technical tools available to mitigate a wide variety of cyber security incidents. However, (advanced) technical tools to support the (internal) working methods of CSIRTs are largely lacking. Technical solutions for exchanging CSIRT related information can be based on standardized technical protocols like STIX, TAXII, and CybOX^3^. The use of the right technical tools that support the work methods can greatly increase the effectiveness of CSIRTs. The effectiveness may lie in the field of lead time of solving the incident, on the financial level and on increasing team knowledge and shared situation awareness within the CSIRT. Tools supporting work methods might include, for example, a tool to estimate the initial impact and risk of a reported cyber security incident in a structured way. The interviews revealed that the initial assessment of the size and risk of a specific cyber security incident is ascertained on an *ad hoc* basis and is predominantly based on the knowledge level of the CSIRT team member who first gets the incident reported.

There is also a need for better intra and inter team communication tools (Fransen and Kerkdijk, 2017). Current tools used as explained in the interviews, such as chat applications, phone calls and wikis, are often inadequate for updating shared awareness within the team, let alone between different teams, especially when there is a need for in-depth technical communication. An adequate communication tool would also support the initial decision to respond to a cyber security incident, for example in the event that information is designated as classified or if communication is necessary with government agencies in a specific format. Ticketing systems are necessary for logging all kinds of events concerning a (possible) cyber security incident but are identified as inadequate for supporting (team)work on larger scale incidents.

Yet another need that was revealed by the interviews is that for tools to provide good and structured reports. For accountability, good and structured written recording of cyber security incidents is indispensable. This implies that during the completion of the mitigation of the cyber security incident, logged events are available and accessible in a user friendly way. It became clear that a lot of logging is done and available, but technical tools to adequately translate this information into good and structured – and reader-friendly – reports is lacking.

Related to the previous need is the desire to be able to create useful (visual) overviews at any certain point in time during an incident for different audiences. Audiences may include the internal management of CSIRTs, the (management of an) organization that is affected by an incident (commercial) business relations, government agencies or even the (public) media. The idea is that visualization tools for providing an overview of different situations during cyber security incident response will improve the understanding of the methods used by the incident handling team and will definitely help adjust the controlling. Applying visualizations will also create better understanding for different audiences for a better insight into the completion of the cyber security incident. The need for the overviews to provide the necessary information in an understandable and accessible way to different audiences implies that the tool must be able to support different levels of detail.

## Discussion

A general finding from the interviews is that there is a great deal of variability in issues that CSIRTs face and in the desires for better team performance. At the same time, it became evident to us that no two CSIRTs are alike. There are many commonalities but also many differences between the CSIRTs we investigated. These differences may be due to several factors, such as type of CSIRT (e.g., internal or commercial provider), the type of organization they work for (e.g., bank, manufacturing company, university, or federal agency), size of the CSIRT and the kind of services they offer. What this implies for an effective solution strategy is that innovations that work for one CSIRT might not work for others. We should keep this in mind as we consider solutions for better practice.

Taken together, if we look at the results, we see a number of mutual needs that can and should be addressed in order to improve CSIRT performance. First, learning from incidents at the organizational level seems to be in need of improvement. Problems were reported with ways to improve performance through systematically implementing a lessons learnt procedure, based on a good evaluation of the incident and how it was managed. This is crucial if CSIRTs are to evolve and structurally improve performance. Further, there is the question of training. This goes not only to keeping abreast of technological developments, but also in regards to softer, social skills such as communication and cooperation.

Second, the coming together of different component teams causes gaps in various manifestations between current and desired incident handling practices. First and foremost, perhaps, it is important to recognize that when different teams come together during a security incident, they are often not much more than a group of teams. In order to become a *team of teams*, in which they function as a multiteam system, they must develop new dynamics and ways of working together. Differences between team cultures hinder this process, as do differences in procedures between the teams’ organizations. Information sharing is a particularly glaring problem in this context. Parties may not know what to share because they do not have a sufficient understanding of what another team needs, or they may not know with whom to share information. Furthermore, ulterior (commercial) motives, may make teams unwilling to share information. It would be interesting to investigate ways to improve the performance at this multiteam level.

Third, improvement of the assessment of the incident in terms of the extent of the problem and the seriousness of the possible consequences is a potential direction for improving performance. It is often difficult to make a good assessment of the incident and coordinate the seeking and synthesizing of data. An interesting solution strategy, in our opinion, is applying knowledge on *collaborative sensemaking* to the incident analysis working processes. Collaborative sensemaking is, basically, the collaborative process of creating shared awareness and understanding out of different individuals’ perspectives in situations of high complexity or uncertainty (Klein et al., 2006). If successful, the outcome of this process is collective understanding of the incident, at which point the proper decision to make is clear or greatly simplified (Klein et al., 2010).

Fourth, characteristics of the work process are candidates for redesign. These include the necessity to hand off work to others and ambiguities or omissions in work procedures. Consider the ethical dilemma in which a team member needs to decide to communicate privacy-sensitive information or the situation when a member needs to decide to scale an incident up or down. In principle, these need not be dilemmas: if the criteria for courses of action are clearly described in procedures, the individual need not be burdened with making these difficult – yet not incident-crucial – decisions on his/her own.

Finally, there is a need for better tools in support of team work. This may be due to unfamiliarity with the existence of certain groupware tools, such as for providing visualizations at the group level. Alternatively, it may be due to resistance to changing the way the teams have always worked, for example when it comes to using tools to estimate size and risk of an incident: this was always done based on team members’ skills and experiences with similar incidents and there is no obvious need to do things differently.

## Conclusion

Computer security incident response teams often have to work under harsh conditions. Teamwork is of the utmost importance and failure due to lack of teamwork could have catastrophic consequences. In this study we set out to find whether there are gaps between current and desired incident handling practices that hinder the performance of these teams. To provide for an effective solution strategy we focused not only on teamwork, but also on gaps at the organizational, individual and instrumental level. Literature review and interviews revealed several gaps, justifying our claim that CSIRTs are at risk to run into problems and that performance could be improved.

There is a high degree of variability in types of CSIRTs, which renders solutions to problems in one type of CSIRT potentially useless for other types of CSIRTs. However, we did identify a number of mutual needs that can and should be addressed in order to improve CSIRT performance regardless of the type of CSIRT. An interesting and promising direction for improvement is applying knowledge on collaborative sensemaking to the incident analysis phase of incident handling. In the incident analysis phase information is sought, combined and reflected upon in order to create ‘sense’ as the basis for further action. This recursive analysis process helps draw useful conclusions from disparate data that vary in accuracy, timeliness, or reliability and validity of sources. Theory of sensemaking helps us to understand how incident analysis works; in other words, what happens during this collaborative process. Sensemaking research has yielded many insights that could be applied to cybersecurity in general and the incident analysis phase in particular.

Several methods have been forwarded to support teams in sensemaking (see, for instance, Veinott et al., 2010). An example is the premortem technique, originally devised by Klein (1998). In premortem, a team, usually a project team at the start of the project, generates plausible reasons for the failure of the plan or project (Veinott et al., 2010). This helps the team to identify risks at the outset, reduce overconfidence and ‘tunnel vision,’ when one tends to focus on a particular conclusion and then to filter all evidence in a case through the lens of that conclusion (Findley and Scott, 2006). Hence, it would be interesting to see to what extent this technique and other insights from collaborative sensemaking research are applicable to the domain of cybersecurity, eventually leading to recommendations for improvements in incident handling practice and beyond.

## Author Contributions

HY and RvdK designed and directed the project. RvdK and GK contacted companies and performed the needs assessment. All authors discussed the results. RvdK wrote the manuscript with support from GK and HY. HY supervised the project.

## Conflict of Interest Statement

The authors declare that the research was conducted in the absence of any commercial or financial relationships that could be construed as a potential conflict of interest.

## Footnotes

1 http://www.cert.org/incident-management/csirt-development/csirt-faq.cfm

2 https://www.government.nl/latest/news/2013/06/21/bill-on-obligation-to-report-data-leakage-sent-to-the-house-of-representatives

3 https://stixproject.github.io/
